# Analysis of key clinical features for achieving complete remission in stage III and IV non-small cell lung cancer patients

**DOI:** 10.1186/s12931-019-1235-3

**Published:** 2019-11-21

**Authors:** Takuya Aoki, Takeshi Akiba, Jun Nishiyama, Sakurako Tajiri, Naoki Hayama, Genki Takahashi, Jun Tanaka, Masako Sato, Hiroto Takiguchi, Hiromi Tomomatsu, Katsuyoshi Tomomatsu, Takahisa Takihara, Kyoko Niimi, Tsuyoshi Oguma, Mitsutomo Kohno, Ryota Masuda, Tetsuya Urano, Hitoshi Itoh, Hiroshi Kajiwara, Naoya Nakamura, Etsuo Kunieda, Mitsunori Matsumae, Masayuki Iwazaki, Koichiro Asano

**Affiliations:** 1 Respiratory Division, Department of Internal Medicine, Tokai University School of Medicine, Isehara, Kanagawa 259-1193 Japan; 2 Department of Radiation Oncology, Tokai University School of Medicine, Isehara, Kanagawa 259-1193 Japan; 3 Department of Neurosurgery, Tokai University School of Medicine, Isehara, Kanagawa 259-1193 Japan; 4 Department of Thoracic Surgery, Tokai University School of Medicine, Isehara, Kanagawa 259-1193 Japan; 5 Department of Pathology, Tokai University School of Medicine, Isehara, Kanagawa 259-1193 Japan

**Keywords:** advanced non-small cell lung cancer, oligometastases, complete remission, radiation, surgery

## Abstract

**Background:**

Although development of immune checkpoint inhibitors and various molecular target agents has extended overall survival time (OS) in advanced non-small cell lung cancer (NSCLC), a complete cure remains rare. We aimed to identify features and treatment modalities of complete remission (CR) cases in stages III and IV NSCLC by analyzing long-term survivors whose OS exceeded 3 years.

**Methods:**

From our hospital database, 1,699 patients, registered as lung cancer between 1^st^ Mar 2004 and 30^th^ Apr 2011, were retrospectively examined. Stage III or IV histologically or cytologically confirmed NSCLC patients with chemotherapy initiated during this period were enrolled. A Cox proportion hazards regression model was used. Data collection was closed on 13^th^ Feb 2017.

**Results:**

There were 164 stage III and 279 stage IV patients, including 37 (22.6%) and 51 (18.3%) long-term survivors and 12 (7.3%) and 5 (1.8%) CR patients, respectively. The long-term survivors were divided into three groups: 3 ≤ OS < 5 years, 5 years ≤ OS with tumor, and 5 years ≤ OS without tumor (CR). The median OS of these groups were 1,405, 2,238, and 2,876 days in stage III and 1,368, 2,503, and 2,643 days in stage IV, respectively. The mean chemotherapy cycle numbers were 16, 20, and 10 in stage III and 24, 25, and 5 in stage IV, respectively. In the stage III CR group, all patients received chemoradiation, all oligometastases were controlled by radiation, and none had brain metastases. Compared with non-CR patients, the stage IV CR patients had smaller primary tumors and fewer metastases, which were independent prognostic factors for OS among long-term survivors. The 80% stage IV CR patients received radiation or surgery for controlling primary tumors, and the surgery rate for oligometastases was high. Pathological findings in the stage IV CR patients revealed that numerous inflammatory cells existed around and inside resected lung and brain tumors, indicating strong immune response.

**Conclusions:**

Multiple line chemotherapies with primary and oligometastatic controls by surgery and/or radiation might achieve cure in certain advanced NSCLC. Cure strategies must be changed according to stage III or IV.

This study was retrospectively registered on 16^th^ Jun 2019 in UMIN Clinical Trials Registry (number UMIN000037078).

## Introduction

The ultimate treatment goal for advanced non-small cell lung cancer (NSCLC) is complete cure. Through the era of chemotherapy as the main treatment option against advanced NSCLC [1–3] for approximately 30 years and even today, various therapeutic strategies have been developed based on a wide range of clinical trials and basic research studies. Consequently, two important new treatments were created: molecular target therapies against driver mutations of oncogenes [4, 5] and immune checkpoint inhibitors (ICI) [6]. The former includes epidermal growth factor receptor-tyrosine kinase inhibitors (EGFR-TKI) [7–9], anaplastic lymphoma kinase-tyrosine kinase inhibitors (ALK-TKI) [10–12], c-ros oncogene 1 (ROS-1) inhibitors [13], v-raf murine sarcoma viral oncogene homolog B1 (BRAF) inhibitors [14], or rearranged during transfection (RET) inhibitors [15, 16]. The latter includes nivolumab [17, 18], pembrolizumab [19, 20], atezolizumab [21] and durvalumab [22]. Discoveries of programmed cell death 1 (PD-1) [23] and cytotoxic T-lymphocyte antigen 4 (CTLA-4) [24] were major breakthroughs, and the disclosure of their roles [25–28] in tumor immune environments has led to the creation of ICI [6, 29, 30], which caused a paradigm shift in cancer treatments. The creation of ICI proved that human immune mechanisms have marked capacity to attack cancers. Owing to this evidence, new innovative treatments are actively being developed. In the near future, combining ICI with other chemotherapies [31, 32] may lead to complete cure in some advanced NSCLC patients, although the development of biomarkers might be crucial to achieving this goal.

On the other hand, controlling oligometastatic disease has been theoretically proposed for curing advanced NSCLC [33], and treatment approaches include stereotactic radiotherapy (SRT), conventionally fractionated radiotherapy, surgery, and radiofrequency ablation (RFA) [34–38]. Clinical trials for oligometastatic diseases have been actively conducted in radiotherapy [39, 40], surgery [41], or both radiotherapy and surgery [42–44], however, optimal treatment approaches, i.e. radiation versus surgery, for the control of oligometastases have not been determined. Moreover, patient selection and the number of metastases, which is regarded as being treatable, have not been established [45]. The superiority of stereotactic ablative radiotherapy as compared to the standard of care palliative treatment was recently demonstrated by a prospective randomized clinical trial [46]. According to this report, three metastases in one organ and a total of 5 metastases were treated. This number might serve as one of the indexes for treatable oligometastases. Although many treatment options and modalities have been developed, the key features of cured patients with advanced NSCLC have not yet been elucidated. Molecular target therapies produce long-term survival, and ICI also prolong survival, a phenomenon known as the tail effect. Complete cure in advanced NSCLC patients empirically occurs very rarely among long-term survivors.

We hypothesized that information on the clinical differences in patient characteristics, lung tumor features and treatment options, including modalities for oligometastatic control, between long-term survivors with and without complete remission (CR), might provide very important clues to achieve complete cure in the treatment of advanced NSCLC. This study was designed to identify the features of advanced NSCLC patients with CR by comparing these factors among long-term survivors whose overall survival time (OS) exceeded 3 years. Complete remission, for the purposes of this study, was defined as sustained CR over the five-year follow-up period since initial treatment, without previous one-year treatment.

## Methods

### Patients

This retrospective study was approved by the institutional review board for clinical research, Tokai University (14R-054). The study was performed in accordance with the Declaration of Helsinki. All patients registered as having lung cancer in our hospital database between 1^st^ Mar 2004 and 30^th^ Apr 2011 were retrospectively examined. The study eligibility criteria for patients were as follows: stage III or IV (The International Association for the Study of Lung Cancer: IASLC 8^th^ edition of the TNM classification for lung cancer) [47], histologically or cytologically confirmed NSCLC, and chemotherapy or EGFR-TKI administration had been initiated between 1^st^ Mar 2004 and 30^th^ Apr 2011. The dataset was closed on 13^th^ Feb 2017, and the final analyses were performed on 10^th^ Aug 2018.

The primary outcome of interest was the OS of all eligible NSCLC patients. The secondary endpoints were patient characteristics, tumor characteristics, and treatment contents and modalities in long-term survivors, whose OS exceeded 3 years, and CR patients. The long-term survivors were divided into three groups: 3 ≤ OS < 5 years (3 ≤ OS < 5), 5 years ≤ OS with tumor (5 ≤ OS tumor +), and 5 years ≤ OS without tumor and no previous one-year treatment (5 ≤ OS tumor -: CR). The features of CR patients were compared among these three groups. OS was defined as elapsed time between the starting date of first chemotherapy or EGFR-TKI administration and the date of death.

### Study design and treatment

This study was a single institutional retrospective study conducted for 13 years. The patients were treated at Tokai University Hospital in Kanagawa, Japan. The treatments were multiple line treatments, and chemotherapies were fully continued according to the American Society of Clinical Oncology (ASCO) guidelines, the National Comprehensive Cancer Network (NCCN) guidelines or the Japan Lung Cancer Society guidelines. Oligometastases, which include oligoreccurences, were considered to control with radiation and/or surgery if patients under various conditions were allowed to receive these modalities and patients hope to receive these treatments.

The extracted data included age, sex, histology, and OS for all enrolled patients, and age, sex, smoking status, body mass index (BMI), Eastern Cooperative Oncology Group (ECOG) performance status (PS), histology, EGFR mutation status, primary tumor diameter, N factor, M factor, total metastatic number, treatment line number, total cycle number, EGFR-TKI usage, EGFR-TKI line number, radiation treatment (primary site and metastatic lesions), surgery (primary and metastatic lesions), treatment of brain metastases (SRT, surgery, and/or whole brain irradiation (WBI)) for long-term survivors. All treatments were examined until death. Total metastatic lesion numbers exceeding 10 and carcinomatous lymphangitis at the time of diagnosis were counted as 10 lesions.

### Statistical analysis

The significance of intergroup differences was determined by the Kruskal-Wallis test, and the Dunn test was used to identify significant differences. The chi-squared test was applied to compare categorical variables. OS was estimated using the Kaplan-Meier method, and median time was reported with 95% confidence intervals (CI). A Cox proportion hazards regression model was used to test the association of each factor with survival time, and a stepwise selection procedure was implemented to identify the independent prognostic roles of patient characteristics. Hazard ratios (HRs) and their 95% CIs were calculated. Differences were considered statistically significant at P < 0.05. All statistical analyses were performed using Stat Flex ver. 6.0 statistical software (Artech Co., Ltd., Osaka, Japan).

## Results

### Enrolled patients

From our hospital database, 1,699 patients were registered as having lung cancer between 1^st^ Mar 2004 and 30^th^ Apr 2011. Among these 1699 patients, 164 stage III and 279 stage IV patients who met the eligibility criteria for this clinical study were enrolled. There were also 144 patients with small cell carcinoma, 287 with early stages who underwent surgery, 113 with only radiation treatment, 109 with best supportive care without chemotherapy, 15 with stage I or II with chemotherapy or chemoradiation, and 588 patients in whom lung cancer could not be confirmed.

The baseline characteristics of the enrolled patients are shown in Table 1. The median age of stage III patients was 68 years (range 41-88), and these patients were older than the stage IV patients (65 years; range 32-89). The proportion of males in the stage III group was larger than that in the stage IV group. Regarding histological types, the proportion of squamous cell carcinoma was highest among stage III patients, while that of adenocarcinoma was highest among stage IV patients.

**Table 1.** Baseline characteristics of all patients with stage III and stage IV non-small cell lung cancers

Variables	Stage III (*n* = 164)	Stage IV (*n* = 279)	*P* value
Age			
Median (Range) – years	68.0 (41-88)	65.0 (32-89)	**< 0.01**
Age distribution			
< 75 – no. (%)	122 (74.4)	232 (83.2)	**< 0.05**
≥ 75 – no. (%)	42 (25.6)	47 (16.8)	
Sex – no. (%)			
Male	137 (83.5)	205 (73.5)	**< 0.05**
Female	27 (16.5)	74 (26.5)	
Histological characteristics of tumor – no. (%)			
Adenocarcinoma	61 (37.2)	200 (71.7)	**< 0.01**
Large cell carcinoma	3 (1.8)	4 (1.4)	
Squamous cell carcinoma	69 (42.1)	34 (12.2)	
Not otherwise specified	26 (15.9)	32 (11.5)	
Other cell types	5 (3.0)	9 (3.2)	

All significant *P* values are expressed in boldface, and not significant *P* values are expressed in plainface

**Fig. 1:**
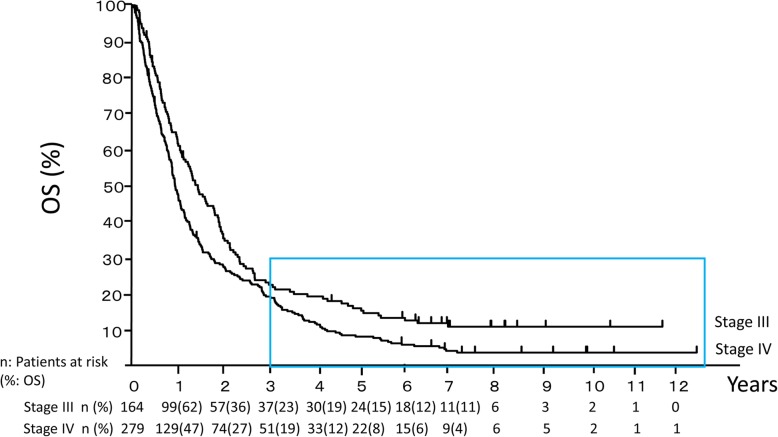
Overall survival (OS) in patients with stage III and IV non-small cell lung cancer. Overall survival was estimated by Kaplan-Meier analysis. Patients inside the square box were long-term survivors, whose OS exceeded 3 years

OS data are shown in Fig. 1. The median OS for stage III patients was 507 days (95% CI; 407-673 days) and 342 days (95% CI; 306-412 days) for stage IV patients. Tail effects were observed in both stage III and stage IV patients.

### Long-term survivors

There were 37 (22.6%) stage III and 51 (18.3%) stage IV long-term survivors. The baseline characteristics of the long-term survivors are shown in Table 2. Although there were significant differences between the stage III and stage IV patients in age, age distribution and sex (Table 1), there were no significant differences in these factors among the long-term survivors (Table 2). There were also no significant differences in smoking status, BMI, EGFR mutation status, ECOG PS, primary tumor diameters and N factor between the stage III and IV long-term survivors. Regarding the histological types of the tumors in long-term survivors, the adenocarcinoma proportion was the highest among both stage III and IV patients.

**Table 2:** Characteristics of the long-term survivors with stage III and stage IV non-small cell lung cancers

Variables	Stage III (*n* = 37)	Stage IV (*n* = 51)	*P* value
Age			
Median (Range) – years	66.0 (41-79)	64.0 (36-89)	0.56
Age distribution			
< 75 – no. (%)	30 (81.1)	43 (84.3)	0.69
≥ 75 – no. (%)	7 (18.9)	8 (15.7)	
Sex – no. (%)			
Male	29 (78.4)	30 (58.8)	0.06
Female	8 (21.6)	21 (41.2)	
Smoking status			
Current	16 (43.2)	19 (37.3)	0.25
Former	13 (35.1)	13 (25.5)	
Never	8 (21.6)	19 (37.3)	
BMI	22.2 ± 3.2	22.2 ± 3.4	0.74
Histological characteristics of tumor – no. (%)			
Adenocarcinoma	20 (54.1)	41 (80.4)	**< 0.05**
Large cell carcinoma	0	1 (2)	
Squamous cell carcinoma	9 (24.3)	3 (5.9)	
Not otherwise specified	6 (16.2)	2 (3.92)	
Other cell types	2 (5.41)	4 (7.84)	
EGFR mutation status Positive/Negative/Unknown	4 / 9 / 24	13 / 13 / 25	0.19
ECOG performance status – no. (%)			
1	17 (46.0)	22 (43.1)	0.79
0	20 (54.1)	29 (56.9)	
Primary tumor diameter (mm; mean ±SD)	36.3 ± 19.3	34.1 ± 15.3	0.91
N factor - no. (%)			
N3	10 (8.11)	15 (29.4)	0.26
N2	22 (59.5)	19 (37.3)	
N1	2 (5.41)	4 (7.8)	
N0	3 (8.11)	13 (25.5)	
M factor - no. (%)			
M1c	0	9 (17.6)	
M1b	0	14 (27.5)	
M1a	0	28 (54.9)	
Total metastatic number at initial diagnosis	0	5.51 ± 4.13	

*BMI* body mass index, *EGFR* epidermal growth factor receptor, *ECOG* Eastern Cooperative Oncology Group.

All significant *P* values are expressed in boldface, and not significant *P* values are expressed in plainface

There were 13 (7.9%), 12 (7.3%), and 12 (7.3%) patients in the 3 ≤ OS < 5, the 5 ≤ OS tumor +, and the CR groups of stage III patients, respectively, and the corresponding median OS for these groups were 1,405 days, 2,238 days, and 2,876 days (Table 3). There were no differences in age, sex, smoking status, BMI, tumor histology, ECOG PS, primary tumor diameter, and N factor. EGFR mutation status was different among the three groups of stage III long-term survivors (Table 3). We also analyzed the influences of occupations/jobs and complications. There were no differences in these parameters among long-term survivors of stage III NSCLC (Table 3).

**Table 3:** Comparison of characteristics among stage III and stage IV long-term survivors according to OS groups

Groups	Stage III				Stage IV			
Variables	3 ≤ OS < 5	5 ≤ OS (tumor +)	5 ≤ OS (tumor -): CR	*P* value	3 ≤ OS < 5	5 ≤ OS (tumor +)	5 ≤ OS (tumor -): CR	*P* value
No. (% of the all stage III or IV patients)	13 (7.9)	12 (7.3)	12 (7.3)		29 (10.4)	17 (6.1)	5 (1.8)	
Median OS	1405	2238**	2876**	**< 0.01**	1368	2503**	2643**	**< 0.01**
(Range) – days	(1097-1770)	(1836-3315)	(2271-4250)		(1109-1791)	(1933-3862)	(2160-4525)	
Age (Median; Range) – yr.	69.0; 50-78	65.5; 54-79	63.5; 41-79	0.47	66; 37-89	67; 41-76	56; 36-69	0.21
Sex-no.								
Male	10	8	11	0.33	14	12	4	0.20
Female	3	4	1		15	5	1	
Smoking status								
Current	4	4	8	0.18	9	6	4	0.15
Previous	4	6	3		6	6	1	
Never	5	2	1		14	5	0	
BMI	21.9 ± 2.97	23.4 ± 4.1	21.2 ± 1.85	0.24	23.0 ± 3.7	21.7 ± 2.5	19.4 ± 2.7	0.10
Complication								
+; no. (%)	11 (85%)	7 (58%)	6 (50%)	0.16	12	7	3	0.73
-; no. (%)	2 (15%)	5 (42%)	6 (50%)		17	10	2	
Type of complication ^#^ – no.				0.74				0.62
COPD	3	4	1		3	0	1	
Pulmonary fibrosis	2	1	0		0	0	0	
Diabetes mellitus	2	3	2		5	1	2	
Heart and aortic diseases	0	2	1		4	1	1	
Previous cancer treatment	3	1	1		0	1	0	
Occupation ^$^				0.56				0.60
Manager, Professional and Technician	2	1	4		8	7	2	
Skilled agricultural, and crafts and related trades	5	4	4		8	6	2	
Others including no regular occupation	6	7	4		13	4	1	
Lymphocyte counts in blood (no. / μl; mean ± SD)	1,785 ± 538	2,249 ± 803	2,182 ± 634	0.29	1,735 ± 503	1,988 ± 521	2,413 ± 710*	**< 0.05**
Histological characteristics of tumor – no. (%)								
Adenocarcinoma	8	8	4	0.37	23	14	4	0.16
Large cell carcinoma	0	0	0		0	0	1	
Squamous cell carcinoma	4	1	4		2	1	0	
Not otherwise specified	1	3	2		2	0	0	
Other cell types	0	0	2		2	2	0	
EGFR mutation status				**0.03**				0.28
Positive/Negative/Unknown	3 / 2 / 8	1 / 6 / 5	0 / 1 / 11		7 / 6 / 16	6 / 4 / 7	0 / 3 /2	
ECOG performance status								
1	5	7	5	0.57	15	7	0	0.10
0	8	5	7		14	10	5	
Primary tumor diameter (mm; mean ± SD)	38.7 ± 22.9	40.0 ± 15.4	30.0 ± 18.6	0.34	39.6 ± 13.6	29.6 ± 14.4	17.5 ± 13.2**	**< 0.01**
N factor – no.								
N3	6	0	4	0.052	13	2	0	**< 0.05**
N2	6	11	5		8	10	1	
N1	0	0	2		3	0	1	
N0	1	1	1		5	5	3	
M factor – no.								
M1c					6	3	0	0.28
M1b					5	6	3	
M1a					18	8	2	
Total metastatic number at initial diagnosis (no.; mean ± SD)	-	-	-		6.6 ± 4.1	5.2 ± 3.8	0.6 ± 0.6**	**< 0.05**

BMI: body mass index, COPD: chronic obstructive pulmonary disease, EGFR: epidermal growth factor receptor, ECOG: Eastern Cooperative Oncology Group, * : *P* < 0.05 and **: *P* < 0.01 compared with the 3 ≤ OS < 5 group. + denotes existence of any complication before treatment. -denotes existence of no complication before treatment. #; Some patients had several complications. $; The International Standard Classification of Occupations (ISCO)-08 was used for this occupational classification. “Not otherwise specified” was based on a pathological determination, with the features being as follows. First, the tumor was pathologically diagnosed as NSCLC. Second, the NSCLC could not be classified into subgroups, due to the lack of positive findings on cytology, hematoxylin-eosin staining and/or immunohistochemical staining

All significant *P* values are expressed in boldface, and not significant *P* values are expressed in plainface

Among the stage IV long-term survivors, there were 29 (10.4%), 17 (6.1%), and 5 (1.8%) patients in the 3 ≤ OS < 5, the 5 ≤ OS tumor +, and the CR groups, respectively, and the median OS for these groups were 1,368 days, 2,503 days, and 2,643 days, respectively (Table 3). There were no differences in age, sex, smoking status, BMI, tumor histology, EGFR mutation status, or ECOG PS. The influences of occupations/jobs and complications were analyzed, and there were no differences in these parameters among long-term survivors of stage IV NSCLC (Table 3).

There were no differences in lymphocyte counts before treatment among the three groups in stage III long-term survivors, while those of the CR group were higher than those of the 3 ≤ OS < 5 group in the stage IV long-term survivors (Table 3). The primary tumor diameters in the CR group were smaller than those in the 3 ≤ OS < 5 group of stage IV patients (Table 3). The N factors in stage IV long-term survivors were different among the three groups, and there were no N3 patients in the CR group with stage IV disease. M factors were not different among the groups of the stage IV long-term survivors, while the total metastatic numbers in the CR group were less than those in the 3 ≤ OS < 5 group of stage IV patients (Table 3).

The univariate and multivariate analyses of the HRs in stage III and stage IV long-term survivors are shown in Table 4. Smoking was associated with decreased HRs in both stage III and stage IV long-term survivors by univariate analysis, while the significance of the HRs for smoking disappeared by multivariate analysis. A univariate analysis of the stage IV long-term survivors revealed increases in the diameter of the primary tumor, and N factor, and metastatic numbers were found to be risk factors for OS. A multivariate analysis of the stage IV long-term survivors revealed increases in BMI, and primary tumor diameter, and total metastatic numbers were independent prognostic factors for OS (Table 4).

**Table 4:** Univariate and multivariate analyses of the hazard ratios (HRs) among Stage III and stage IV long-term survivors

	Stage III				Stage IV			
	Univariate		Multivariate		Univariate		Multivariate	
	HR (95% CI)	*P* value	HR (95% CI)	*P* value	HR (95% CI)	*P* value	HR (95% CI)	*P* value
Age	1.03 (0.98-1.08)	0.29	1.00 (0.94-1.06)	0.91	1.01 (0.99-1.04)	0.29	1.00 (0.98-1.03)	0.74
Sex								
Male	0.73 (0.26-2.03)	0.54	1.27 (0.38-4.19)	0.70	0.65 (0.35-1.21)	0.17	1.00 (0.41-2.42)	0.99
Female (ref)								
Smoking status								
Current	0.28 (0.09-0.89)		0.23 (0.04-1.22)		0.47 (0.23-0.99)		0.36 (0.13-1.01)	
Previous	0.53 (0.29-0.94)	**0.03**	0.48 (0.21-1.11)	0.09	0.69 (0.48-0.995)	**0.047**	0.60 (0.36-1.01)	0.052
Never (ref)								
ECOG performance status								
1	0.63 (0.25-1.60)	0.33	0.96 (0.31-2.94)	0.94	1.75 (0.94-3.24)	0.08	1.45 (0.70-3.01)	0.31
0 (ref)								
BMI	1.03 (0.91-1.17)	0.62	1.02 (0.91-1.15)	0.73	1.10 (0.99-1.21)	0.07	1.14 (1.01-1.29)	**0.04**
Primary tumor diameter (mm)	1.02 (0.99-1.04)	0.18	1.03 (0.99-1.06)	0.11	1.04 (1.02-1.07)	**< 0.01**	1.04 (1.02-1.07)	**< 0.01**
N factor								
N3	2.32 (0.34-15.9)		1.77 (0.13-23.7)		3.50 (1.33-9.20)		1.87 (0.59-5.93)	
N2	1.75 (0.49-6.33)		1.46 (0.26-8.25)		2.31 (1.21-4.39)		1.52 (0.70-3.28)	
N1	1.32 (0.70-2.52)	0.39	1.21 (0.51-2.87)	0.67	1.52 (1.10-2.10)	**0.01**	1.23 (0.84-1.81)	0.29
N0 (ref)								
M factor	-		-					
M1c					0.87 (0.37-2.04)		0.74 (0.30-1.85)	
M1b					0.93 (0.61-1.43)	0.74	0.86 (0.55-1.36)	0.52
M1a (ref)								
Total metastatic number at initial diagnosis	-		-		1.09 (1.01-1.18)	**0.03**	1.11 (1.002-1.23)	**0.045**

*HR* Hazard ration, *ref* reference.

All significant *P* values are expressed in boldface, and not significant *P* values are expressed in plainface

### Treatment

The treatment differences among the groups of stage III and IV long-term survivors are shown in Table 5. The treatment line number in the CR group of stage III patients was less than that in the 3 ≤ OS < 5 group, indicating that CR patients did not need many lines of chemotherapy to achieve CR. There was no difference in EGFR-TKI usage among the groups of stage III long-term survivors. Radiation treatment for the primary site was performed in 85%, 67%, and 100% of cases in the 3 ≤ OS < 5, the 5 ≤ OS tumor +, and the CR groups, respectively, of stage III long-term survivors. All stage III CR patients received chemoradiation treatment. Three stage III CR patients (25%) received radiation treatment for metastatic lesions other than brain metastases. No stage III CR patients received surgery for primary or metastatic lesions. No patients received treatments for brain metastases in the stage III CR group because no brain metastases developed in this group.

**Table 5:** Treatment differences among the groups of stage III and stage IV long-term survivors

Variables	Stage III				Stage IV			
Groups	3 ≤ OS < 5	5 ≤ OS (tumor +)	5 ≤ OS (tumor -): CR	*P* value	3 ≤ OS < 5	5 ≤ OS (tumor +)	5 ≤ OS (tumor -): CR	*P* value
Chemotherapy								
Treatment line No. (mean ± SD)	3.7 ± 2.0	3.7 ± 2.8	1.7 ± 1.0*	**< 0.05**	5.9 ± 2.5	5.5 ± 3.4	2.0 ± 1.4*	**< 0.05**
Total cycle No. (include EGFR-TKI#) (mean ± SD)	16.3 ± 10.2	20.3 ± 19.6	10.2 ± 11.7	0.20	23.8 ± 13.6	25.1 ± 18.4	5.4 ± 3.5*^#^	**< 0.05**
EGFR-TKI usage								
+; no. (%)	4 (31%)	3 (25%)	0 (0%)	0.12	20 (69%)	11 (65%)	1 (20%)	0.11
-; no. (%)	9 (69%)	9 (75%)	12 (100%)		9 (31%)	6 (35%)	4 (80%)	
EGFR-TKI line No.	0.5 ± 0.8min: 1, max: 2	0.5 ± 1.0 min: 1, max: 3	0	0.13	1.5 ± 1.3 min: 1, max: 4	1.1 ± 1.1 min: 1, max: 3	0.4 ± 0.9 min: 2, max: 2	0.15
Radiation								
Primary								
+; no. (%)	11 (85%)	8 (67%)	12 (100%)	0.09	5 (17%)	4 (24%)	2 (40%)	0.51
-; no. (%)	2 (15%)	4 (33%)	0 (0%)		24 (83%)	13 (76%)	3 (60%)	
Metastatic lesions other than brain								
+; no. (%)	1 (8%)	3 (25%)	3 (25%)	0.43	11 (38%)	5 (29%)	2 (40%)	0.82
-; no. (%)	12 (92%)	9 (75%)	9 (75%)		18 (62%)	12 (71%)	3 (60%)	
Both primary and metastatic lesions other than brain								
+; no. (%)	1 (8%)	3 (25%)	3 (25%)	0.43	2 (7%)	1 (6%)	0 (0%)	0.83
-; no. (%)	12 (92%)	9 (75%)	9 (75%)		27 (93%)	16 (94%)	5 (100%)	
Surgery								
Primary								
+; no. (%)	1 (8%)	3 (25%)	0 (0%)	0.13	2 (7%)	1 (6%)	2 (40%)	0.06
-; no. (%)	12 (92%)	9 (75%)	12 (100%)		27 (93%)	16 (94%)	3 (60%)	
Metastatic lesions other than brain								
+; no. (%)	0 (0%)	0 (0%)	0 (0%)	1.00	0 (0%)	0 (0%)	2 (40%)	**< 0.05**
-; no. (%)	13 (100%)	12 (100%)	12 (100%)		29 (100%)	17 (100%)	3 (60%)	
Both primary and metastatic lesions other than brain								
+; no. (%)	0 (0%)	0 (0%)	0 (0%)	1.00	0 (0%)	0 (0%)	1 (20%)	**< 0.05**
-; no. (%)	13 (100%)	12 (100%)	12 (100%)		29 (100%)	17 (100%)	4 (80%)	
Treatment of brain metastases								
SRT								
+; no. (%)	1 (8%)	3 (25%)	0 (0%)	0.13	8 (28%)	5 (29%)	1 (20%)	0.92
-; no. (%)	12 (92%)	9 (75%)	12 (100%)		21 (72%)	12 (71%)	4 (80%)	
Surgery								
+; no. (%)	0 (0%)	0 (0%)	0 (0%)	1.00	1 (3%)	1 (6%)	1 (20%)	0.35
-; no. (%)	13 (100%)	12 (100%)	12 (100%)		28 (97%)	16 (94%)	4 (80%)	
WBI								
+; no. (%)	1 (8%)	1 (8%)	0 (0%)	0.60	7 (24%)	3 (18%)	0 (0%)	0.44
-; no. (%)	12 (92%)	11 (92%)	12 (100%)		22 (76%)	14 (82%)	5 (100%)	

#: One continuous EGFR-TKI usage is considered as 1 cycle, *: < 0.05 compared with the 3 ≤ OS < 5 group, ^#^: < 0.05 compared with the 5 ≤ OS with tumor (tumor +) group, + denotes done of the treatment. -denotes not-done of the treatment. Metastatic lesions include at initial diagnosis and during treatment course.

All significant *P* values are expressed in boldface, and not significant *P* values are expressed in plainface

The treatment line number in the CR group of stage IV long-term survivors was also less than that in the 3 ≤ OS < 5 group. There was no difference in the proportion of EGFR-TKI usage. In the stage IV CR group, 2 patients received radiation therapy for the primary tumor, and 2 patients received radiation therapy for metastatic lesions other than brain metastases, while no patients received radiation treatments for both sites. Two patients received surgery for the primary site, 2 patients received surgery for metastatic lesions other than brain metastases, and 1 patient received surgery for both primary and metastatic lesions. Four out of the 5 CR patients received radiation (2 patients) or surgery (2 patients) for the primary tumor. The rates of surgery for metastatic lesions other than brain metastases and for both primary and metastatic lesions were different among the groups (Table 5), showing that the rate of surgery for controlling oligometastases was high in the stage IV CR group. One patient in the stage IV CR group received both SRT and brain surgery for brain metastases. No patients in the stage IV CR group were treated with WBI.

**Fig. 2:**
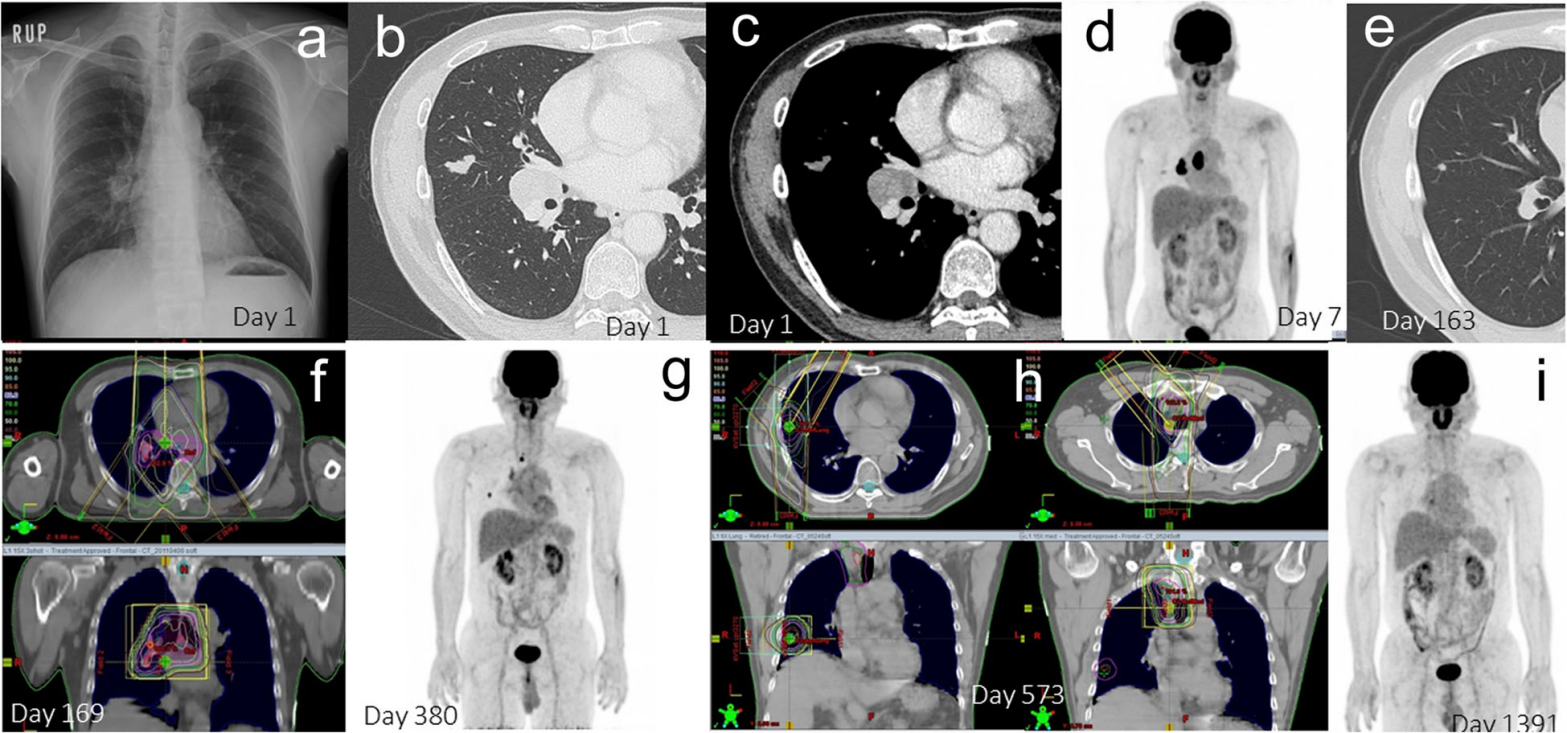
A representative complete remission (CR) patient with oligometastases in stage III. A CR patient with stage III (**a**-**i**), in whom successful control of primary and oligometastatic lesions was obtained, is shown. A male in his 50s consulted our hospital due to an abnormal shadow recognized on a chest X-ray (**a**). The computed tomography (CT) revealed the primary lesion to be in the right middle lobe (**b**), as well as swelling of a right hilar (**c**) and a mediastinal lymph node. Positron emission tomography (PET) / CT (**d**) and head magnetic resonance imaging (MRI) demonstrated the patient to have stage IIIA (cT1bN2M0) adenocarcinoma. The primary lesion showed scar formation after chemotherapy (**e**). However, PET/CT showed viable cells in the right hilar lymph node. Three-dimensional conformal radiation therapy was planned for the right hilar and mediastinal lymph nodes (**f**). On the day 380 since initial treatment, PET/CT revealed recurrence of the primary lesion and a new mediastinal lymph node metastasis (**g**). Three-dimensional conformal radiation therapy was administered for these lesions (**h**). The PET/CT (**i**) and the head MRI demonstrated CR

**Fig. 3:**
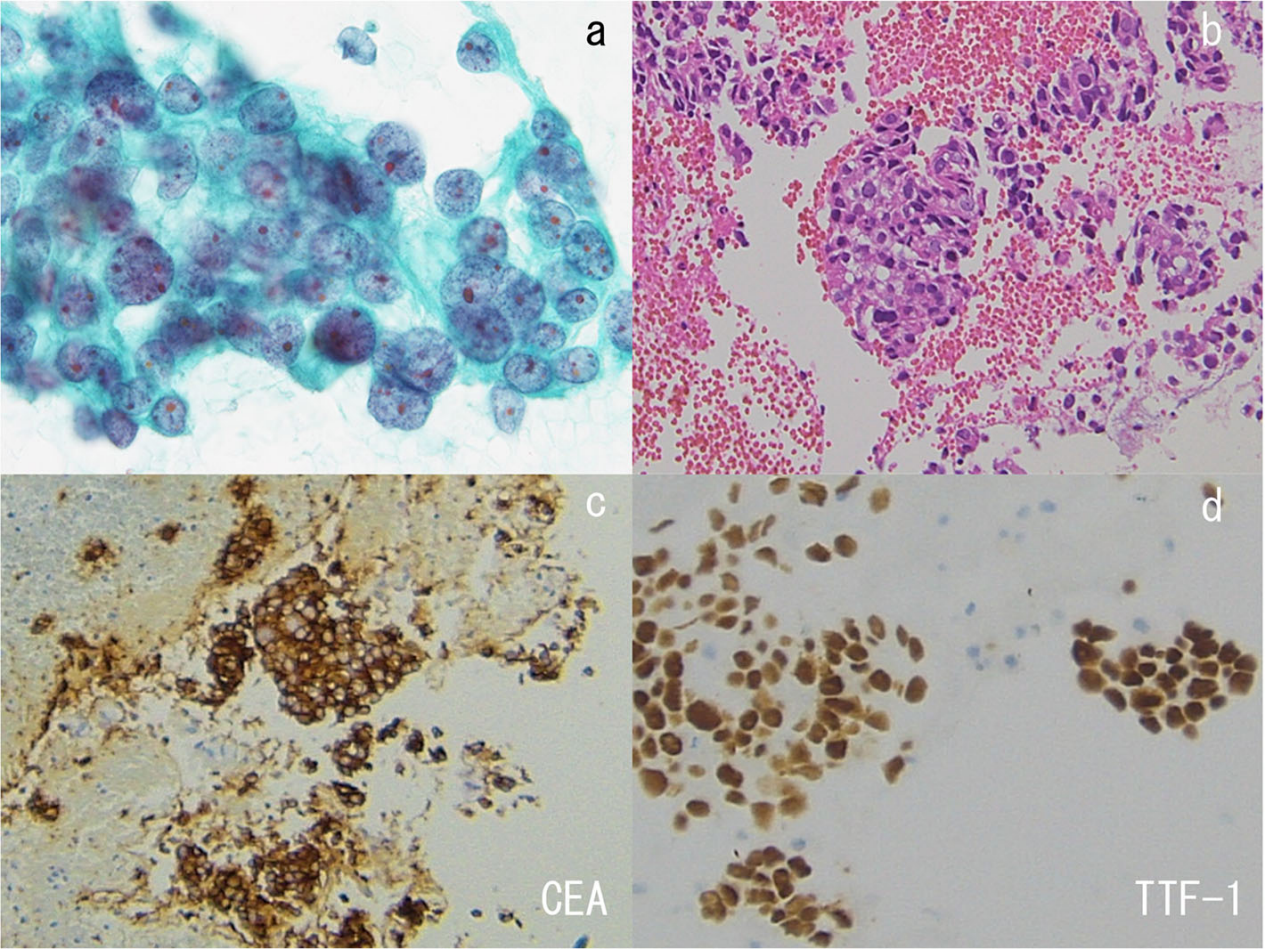
Pathological findings in the stage III patient with complete remission (CR), shown in Fig. 2. Cytology by bronchoscopy was class V according to the Papanicolaou staining (**a**). Histological examinations confirmed the diagnosis of adenocarcinoma, based on hematoxylin and eosin staining (**b**) and immunohistochemical staining for CEA (**c**) and TTF-1 (**d**). The carcinoma cells were positive for CEA (**c**) and TTF-1 (**d**)

A representative CR patient with oligometastatic control in stage III is shown in Fig. 2. The patient was treated with chemoradiation, and oligometastatic states were successfully controlled by positron emission tomography / computed tomography (PET/CT)-assisted 3 dimensional conformal radiation therapy. A male in his 50s, who was referred to our hospital due to an abnormal shadow noted on a chest X-ray (Fig. 2a), was diagnosed as stage IIIA (cT1bN2M0) adenocarcinoma (Fig. 2a-d, Fig. 3). Serum carcinoembryonic antigen (CEA) was 553.2 ng/ml. The primary lesion was in the right middle lobe (Fig. 2b). Swelling of a right hilar and mediastinal lymph node was noted (Fig. 2c and Fig. 2d). This patient received a chemotherapeutic regimen with carboplatin + paclitaxel + bevacizumab. The primary lesion showed scar formation (Fig. 2e). However, PET/CT showed viable cells in a right hilar lymph node. Three-dimensional conformal radiation therapy was planned for the right hilar and mediastinal lymph nodes (Fig. 2f). The total radiation dose was 60Gy. At 380 days after the initial treatment, fluorodeoxyglucose (FDG) accumulations at the primary lesion and a newly-involved mediastinal lymph node (#3) were observed by PET/CT scan (Fig. 2g). Three-dimensional conformal radiation therapy for these lesions (total, 62.5Gy for each) was administered to control these lesions (Fig. 2h). This patient achieved CR (Fig. 2i), has maintained this CR over 5 years since initial treatment without previous one-year treatment.

**Fig. 4:**
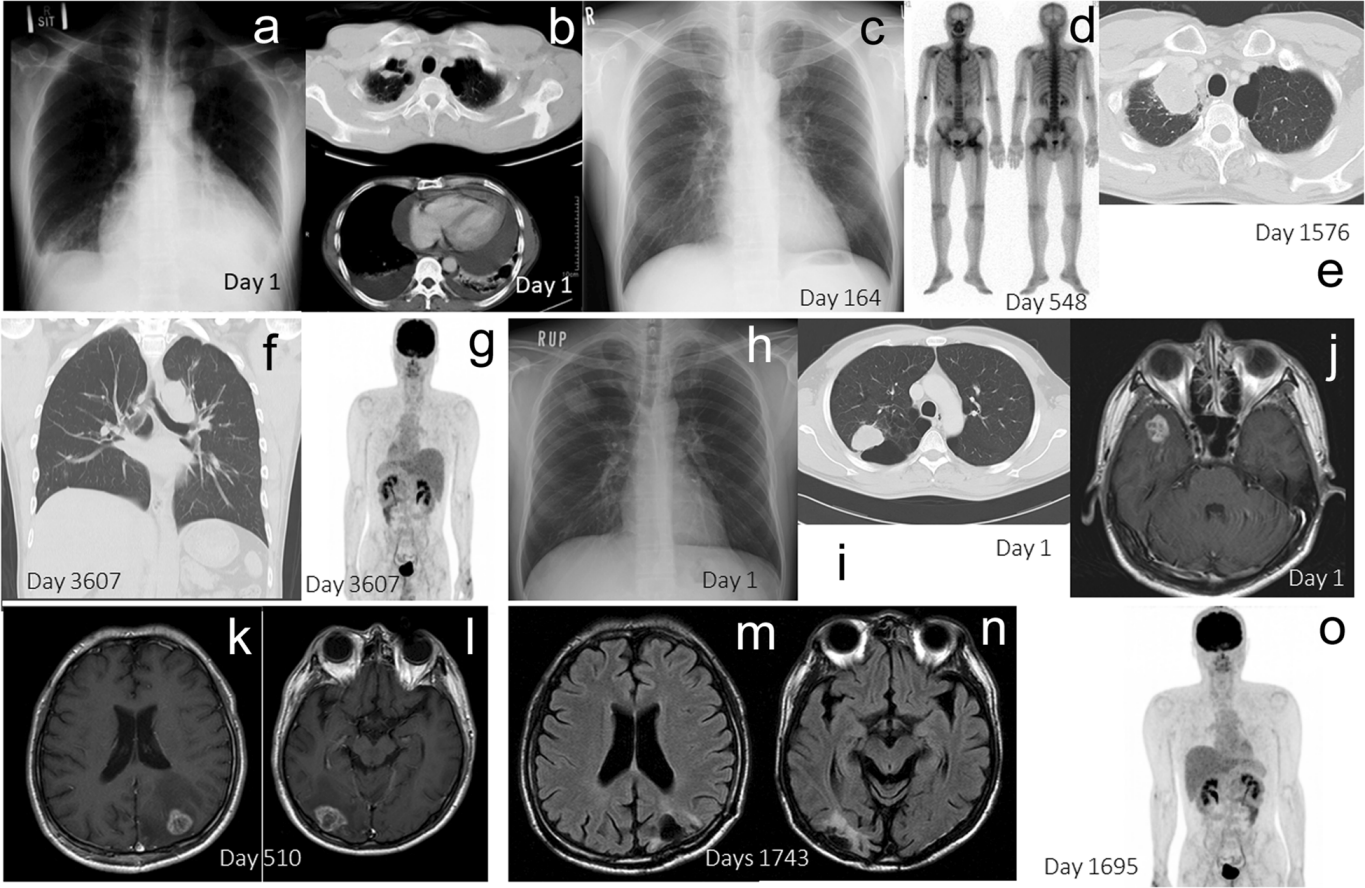
Two representative complete remission (CR) patients with oligometastases in stage IV. The first CR patient in stage IV is shown in a-g. A male in his 50s presented with dyspnea, and bilateral malignant pleural and pericardial effusions were observed on the chest X-ray (**a**) and the CT (**b**). He was diagnosed as stage IVA (cT1aN2M1a) adenocarcinoma. After pericardial drainage followed by chemotherapy, the pleural and pericardial effusions disappeared (**c**). He complained of left femoral pain, and bone scintigraphy revealed bone metastases in the left femoral bone and left hip joint (**d**). After radiation therapy, he underwent joint replacement surgery. The primary lesion, located in the right upper lobe, showed gradual enlargement (**e**) and was surgically removed (**f**). The CR has since been maintained, based on head MRI and PET/CT (**g**). The second patient with brain metastases achieving CR is shown in h-o. A male in his 30s consulted our hospital due to loss of consciousness. A tumor was detected in the right upper lobe (**h**, **i**). There was no lymph node swelling, while a solitary brain metastasis was observed in the right temporal lobe (**j**). He was diagnosed as stage IVA (cT2aN0M1b) large cell carcinoma. Cyber-knife therapy for this lesion and surgery for the primary lesion were performed. After 4 courses of a first-line chemotherapy, three new brain metastases were detected in the left cerebellum, the right occipital lobe, and the left parietal lobe by head MRI. Cyber-knife treatments for these lesions were done. He subsequently complained of headache. Brain metastases in the left occipital lobe (**k**), the left cerebellum, and the right occipital lobe (**l**) were demonstrated by head MRI. Brain surgeries were performed for these lesions. The brain tumors and the lung tumor ultimately disappeared, as shown by the head MRI (**m**, **n**) and the PET/CT (**o**)

**Fig. 5:**
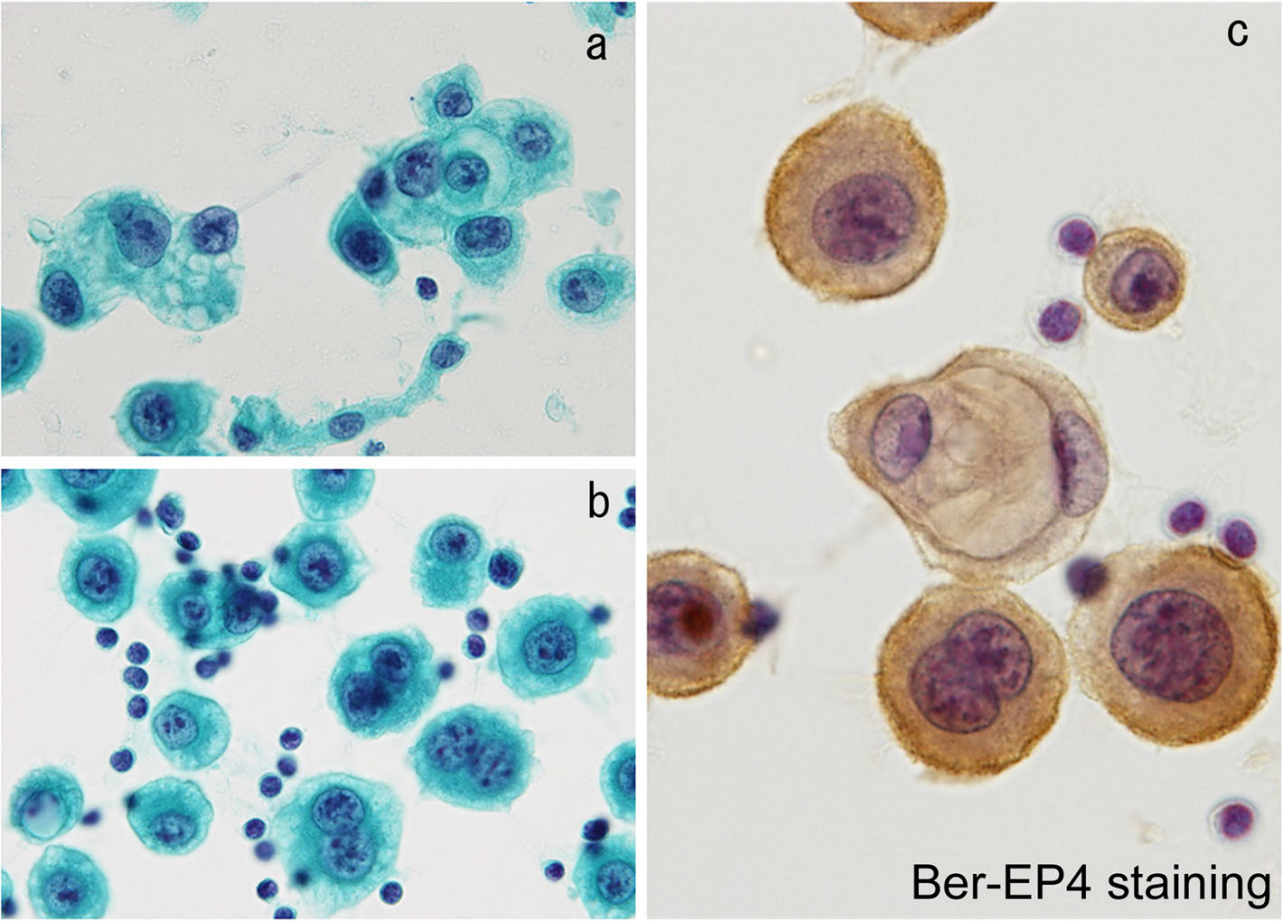
Cytological findings in a stage IV patient with complete remission (CR), shown in Fig. 4a-g. Carcinoma cells were confirmed by Papanicolaou staining of pericardial effusion (**a**, **b**). This carcinoma was a very aggressive type, based on the presence of numerous mitotic cells (**b**). Carcinoma cells stained positive for Ber-EP4 (**c**), indicating these cells to be adenocarcinoma, not mesothelioma

**Fig. 6:**
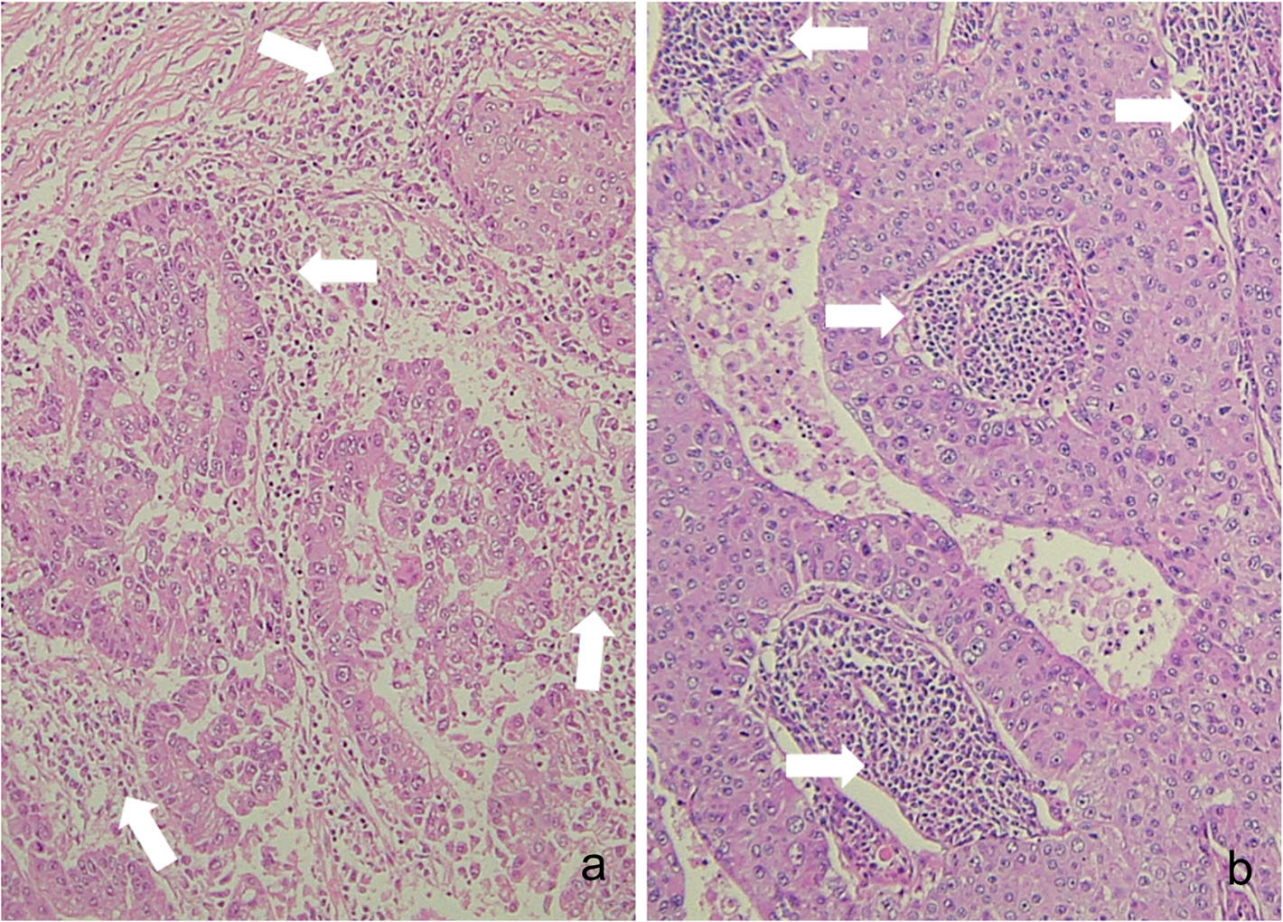
Pathological findings of the lung tumor resected from the right upper lobe, shown in Fig. 4e. Hematoxylin and eosin staining of the resected tumor is shown. Pathological examination revealed this tumor to be an adenocarcinoma showing a mixture of the acinar type (**a**) and the solid type (**b**). Numerous inflammatory cells around (**a**) and inside (**b**) the tumor are indicated by the white arrows. A lot of lymphoid follicles are visible inside the tumor (**b**). These inflammatory cells demonstrate marked immune response

Two representative CR patients with stage IV NSCLC, who received oligometastatic treatments with radiation and surgery, are shown in Fig. 4a-g and Fig. 4h-o. The first patient was treated with a chemotherapeutic regimen followed by EGFR-TKI, and the oligometastatic lesions were controlled with radiation and surgery (Fig. 4a-g). A male in his 50s consulted our institution for dyspnea, and was hospitalized due to bilateral malignant pleural effusion, malignant pericardial effusion and cardiac tamponade (Fig. 4a and Fig. 4b). He was diagnosed as Stage IVA (cT1aN2M1a) adenocarcinoma (Fig. 5). Biopsy of an enlarged right-sided lower pretracheal lymph node (#3) confirmed N2. After pericardial drainage, a chemotherapeutic regimen with cisplatin + gemcitabine was administered. After 4 courses of cisplatin + gemcitabine treatment, the pleural and pericardial effusions disappeared (Fig. 4c). Gefitinib was started. He complained of left femoral pain. Metastases were demonstrated by bone scintigraphy in the left femoral bone and left hip joint on day 548 (Fig. 4d). After the radiation therapy (total 45Gy: 3 Gy/fraction x 15 fractions) to the left femoral area, joint replacement surgery was performed. The primary lesion gradually enlarged thereafter (Fig. 4e), and the mass in the right upper lobe of the lung was surgically removed (Fig. 4f). Pathological examination revealed this tumor to be an adenocarcinoma (Fig. 6) showing a mixture of the acinar subtype (Fig. 6a) and the solid type (Fig. 6b). The EGFR mutations were, however, negative. There were no mutations of EGFR exon 18 G719X, exon 21 L858R, exon 21 L861Q, exon 19 deletion, and exon 20 T790M. The patient was also negative for the Alk mutation based on the immunohistochemistry and fluorescence *in situ* hybridization (FISH) methods. Numerous inflammatory cell infiltration was observed around the tumor (Fig. 6a), and many lymphoid follicles were also detected in the resected lung tumor (Fig. 6b), demonstrating strong immune responses against the tumor. The CR has been maintained (Fig. 4f and Fig. 4g) over 5 years since initial treatment without previous one-year therapy.

**Fig. 7:**
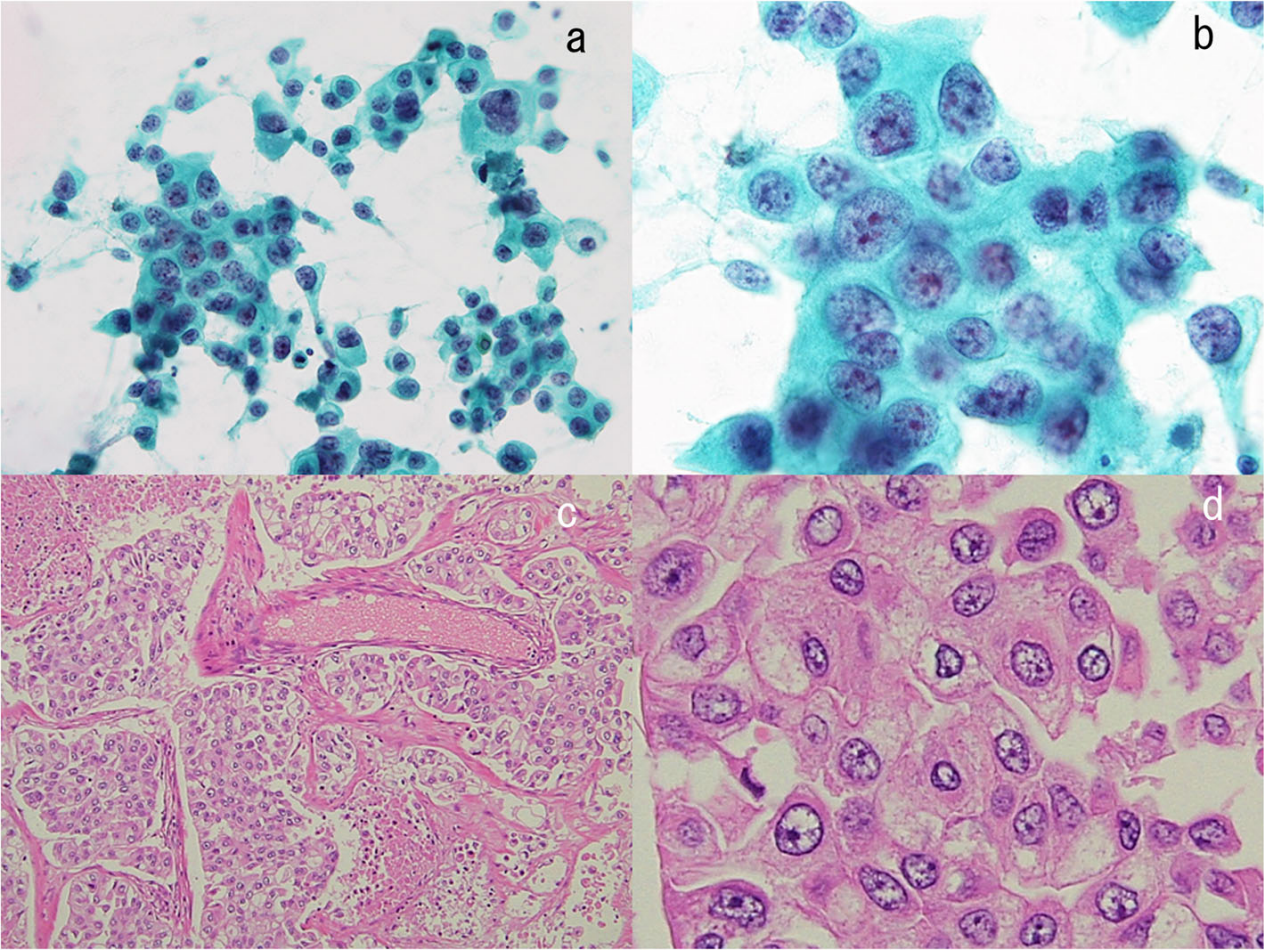
Pathological findings in a stage IV patient with complete remission (CR), shown in Fig. 4h and i. Bronchoscopy was performed. Cytological examination revealed non-small cell type carcinoma, according to Papanicolaou staining (low power field; **a**, high power field; **b**). The surgically resected primary lung tumor (Fig. 4h and i) was stained with hematoxylin and eosin (low power field; **c**, high power field; **d**). Large cell carcinoma was confirmed by pathological examination

**Fig. 8:**
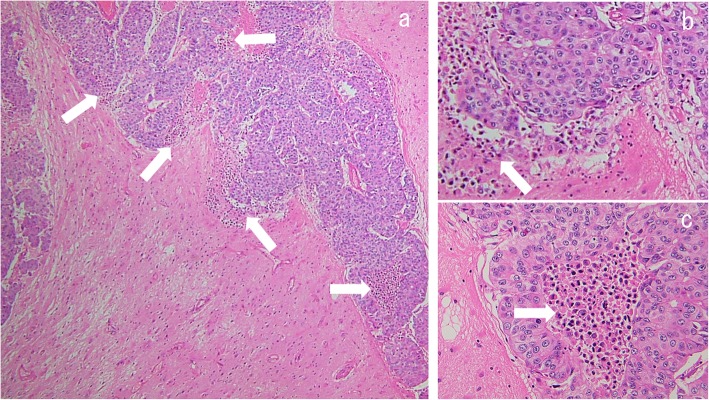
Pathological findings of the resected brain tumor in the right occipital lobe in Fig. 4n. This slide shows hematoxylin and eosin staining of the lesion resected from the right occipital lobe (low power field; **a**, high power field; **b** and **c**). Metastatic large cell carcinoma was confirmed. There are inflammatory cells around (**b**) and inside (**c**) the carcinoma, indicating strong immune response

Another stage IV CR patient with brain metastases is shown in Fig. 4h-o. He was treated with multiple chemotherapeutic regimens and primary surgical resection, and the brain metastases were controlled with SRT and brain surgeries. This male, in his 30s, consulted due to loss of consciousness. He was diagnosed as stage IVA (cT2aN0M1b) large cell carcinoma (Fig. 4h-i, Fig. 7) with a solitary brain metastasis (Fig. 4j). Cyber-knife therapy for the brain tumor was done. We planned to start chemotherapy, however, he developed pneumothorax. The operation on the primary lesion was decided before the initiation of chemotherapy. After 4 courses of a first-line chemotherapy regimen with cisplatin + docetaxel, three new brain metastases were observed in the left cerebellum, the right occipital lobe, and the left parietal lobe by head magnetic resonance imaging (MRI). Cyber-knife treatments to these three new brain metastases were done. He complained of headache. A new lesion in the left occipital lobe was observed (Fig. 4k), and the tumors in the left cerebellum and the right occipital lobe (Fig. 4l) were getting large. Brain surgery was undertaken. Resected brain tumor in the right occipital lobe is shown in Fig. 8. Numerous inflammatory cell infiltration was observed around (Fig. 8a and b) and within (Fig. 8a and c) the metastatic brain carcinoma, showing marked immune response. Finally, the brain tumors and the lung tumor disappeared (Fig. 4m-o). He has maintained CR for more than 5 years without previous one-year treatment.

Four tumors and one cranial dura mater specimen from two stage IV CR patients were histopathologically examined. Three tumors and one cranial dura mater specimen were obtained from the brain metastases of the patient presented in Fig. 4h-o, and also one pulmonary lung tumor from the patient shown in Fig. 4a-g. Strong inflammatory responses, which were confirmed by abundant inflammatory cell infiltrations, were observed in all of these lesions.

## Discussion

This study, conducted in Japan, is the first to explore the features in CR cases of advanced NSCLC, including both stage III and stage IV. Among stage III patients, all CR patients received chemoradiation therapy, and no brain metastases occurred during the treatments without prophylactic WBI (Table 5). Distant metastases during the course of treatment, which are considered as oligometastases or oligorecurrence, were radically controlled by radiotherapies, not surgeries (Table 5). On the other hand, among stage IV patients, there were also CR patients with different characteristics from those of stage III patients (Table 3, Table 4, Table 5). The stage IV CR patients had smaller primary tumors and fewer metastases (Table 3, Table 4). The mean diameter was 17.5 ± 13.2 mm, and the mean metastatic number was 0.6 ± 0.6, indicating that these patients were stage IV with one distant metastasis or malignant pleural or pericardial effusion at the initial diagnosis, and there were no N3 patients. Even though smaller primary tumors and fewer metastases, treatments with surgery or radiation combined with systemic chemotherapies were necessary to control the primary and metastatic lesions, suggesting the difficulty of curing stage IV NSCLC patients.

The clinical characteristics of the CR patients were quite different between stage III and stage IV groups, showing that lung tumors have different biological identities between the two stages. Hence, we must possibly change the strategies for achieving complete cure according to whether NSCLC is stage III or stage IV. The features of the stage III CR patients in this study suggest that the active radical control of all oligometastases with radiation might be effective for inducing CR after chemoradiation, if brain metastases do not occur. Treatments with chemoradiation followed by durvalumab combined with the radical control of oligometastases are expected to increase cure rates in stage III patients, because a low incidence of new brain metastases with durvalumab was reported in the PACIFIC study [22], a hypothesis that requires confirmation in future clinical trials. Smoking is associated with tumor mutation burden and EGFR mutation status. Increased smoking, especially, induces high mutation burden in NSCLC, which might be attributable to decreased HRs in long-term survivors receiving continuous treatment in both stage III and stage IV, as demonstrated by univariate analysis (Table 4). There may be multiple confounding factors, due to which the HRs for smoking might disappear on multivariate analysis (Table 4).

Owing to the development of ICI with platinum doublet chemotherapy as a first-line treatment, a higher response rate and more extended OS were reported in phase 3 trials for both nonsquamous [48, 49] and squamous metastatic NSCLC [50]. The Keynote 189 clinical trial, which targeted metastatic nonsquamous NSCLC, reported that the estimated proportion of patients who were alive at 12 months was 69.2% in the pembrolizumab-pemetrexed-platinum group [48], and the IMpower 150 clinical trial also targeted metastatic nonsquamous NSCLC, and the median OS was reported as 19.2 months in the atezolizumab-bevacizumab-paclitaxel-platinum group [49]. The Keynote 407 clinical trial, which targeted metastatic squamous NSCLC, reported that the median OS was 15.9 months in the pembrolizumab-paclitaxel-platinum group [50]. Compared to those of previously reported phase 3 clinical trials, these recent results are surprisingly effective for the treatment of stage IV NSCLC. Moreover, in locally advanced unresectable stage III NSCLC, chemoradiation with ICI induced a higher response rate and longer median time to death or distant metastases, which were 28.4% and 23.2 months, respectively, in the durvalumab group in the PACIFIC study [22]. Taken together, in the treatment of both stage III and stage IV NSCLC, long-term survivors are obviously increased by adding ICI to the conventional standard treatments. However, cure strategies have not yet been developed. Therefore, we aimed to obtain clues for complete cure in this study. We considered a cure as sustained tumor disappearance over the 5-year period since initial chemotherapy or EGFR-TKI initiation, with no treatment for previous one year, which was defined as CR in this study.

In the resected tumors in the stage IV CR patients, abundant inflammatory cell infiltration was observed. The blood lymphocytes before treatment were also increased in the stage IV CR patients (Table 3). These findings, taken together with small tumors and few metastases, raise the following two possibilities in cured patients. First, host immune systems prevent tumor growth and the occurrence of metastases. Second, the growth speed of the tumor is low, and the ability of metastases is also low. In other words, if systemic treatments with ICI and/or chemotherapies control primary and metastatic sites for a long time, considered as a dormant state, active radical therapies for the remaining lesions might be expected to increase the cure rates of stage IV patients. Endo et al. [41] reported a similar conclusion, in a prospective study of 34 patients, showing that clinical T1-2 N0-1 lung cancer with a single-organ metastatic lesion was a good candidate for surgical resection, and a 5-year survival rate of approximately 40% can be expected. The prognostic importance of tumor-infiltrating lymphocytes and their potential predictive significance were recently reported in solid tumors including NSCLC [51]. Researches focusing on tumor-infiltrating lymphocytes are anticipated to yield further insights into their roles and functions in a tumor microenvironment.

By using the Kaplan-Meier method, the estimated OS in our study at the 3-year time point was 19% for stage IV NSCLC patients (Fig. 1). The results of this study, obtained from chemotherapy without ICI, are comparable to the reported OS with ICI monotherapy. In the pooled analysis of the CheckMate 017 and CheckMate 057 phase III clinical trials, which targeted stage IIIB/IV squamous cell carcinoma and stage IIIB/IV nonsquamous cell carcinoma, respectively, the estimated OS at the 3-year time point was 17% [52]. Continuous treatments with chemotherapies without ICI are suggested to have similar effects as ICI monotherapies, although the similarity was not statistically confirmed.

There are several limitations of this study. This study was a single institutional retrospective study, and future prospective multi-institutional clinical studies are necessary based on the results of this study. The statistical power to compare the groups might be low owing to the small number of patients, because CR patients are not common, particularly in stage IV. Lymphocytes might play a major role as immune cells, because neutrophils or myeloid derived suppressor cells are also abundant in NSCLC tissue and do not have beneficial effects on survival. Strong immune responses are shown in the tumor tissues derived from the stage IV CR patients, while we could not precisely distinguish individually these tumor-infiltrating cells. Nevertheless, we believe that the results of this analysis provide effective clues for developing further clinical trials to cure advanced NSCLC.

## Conclusion

In summary, this retrospective study demonstrated that CR are possible in certain patients by controlling primary lesions and oligometastases through surgery and/or radiation combined with chemotherapies, even in advanced NSCLC. All stage III CR patients received chemoradiation therapy with oligometastatic control by radiation therapies, and no brain metastases occurred during the treatment course. On the other hand, stage IV CR patients had smaller primary tumors and fewer metastases among the long-term survivors, there were no N3 patients, and marked inflammatory cell infiltration was observed in the surgically resected lung and brain tumors, suggesting the importance of host immune response against lung cancer.

## Data Availability

The dataset used and/or analyzed during the current study are available from the corresponding author on reasonable request.

## Abbreviations

ALK-TKI: anaplastic lymphoma kinase-tyrosine kinase inhibitors
ASCO: The American Society of Clinical Oncology
BMI: body mass index
BRAF: v-raf murine sarcoma viral oncogene homolog B1
CEA: carcinoembryonic antigen
CI: confidence intervals
CR: complete remission
CTLA-4: cytotoxic T-lymphocyte antigen 4
ECOG: Eastern Cooperative Oncology Group
EGFR-TKI: epidermal growth factor receptor-tyrosine kinase inhibitors
FDG: fluorodeoxyglucose
FISH: fluorescence *in situ* hybridization
HRs: hazard ratios
IASLC: The International Association for the Study of Lung Cancer
ICI: immune checkpoint inhibitors
MRI: magnetic resonance imaging
NCCN: The National Comprehensive Cancer Network
NSCLC: non-small cell lung cancer
OS: overall survival time
PD-1: programmed cell death 1
PET/CT: positron emission tomography / computed tomography
PS: performance status
RET: rearranged during transfection
RFA: radiofrequency ablation
ROS-1: c-ros oncogene 1
SRT: stereotactic radiotherapy
UMIN: university hospital medical information network
WBI: whole brain irradiation
